# Social skills interventions for Thai adolescents with Autism Spectrum Disorder (ASD): a qualitative study of the perceptions and experiences of Thai adolescents, their caregivers and healthcare professionals

**DOI:** 10.1186/s13033-023-00617-3

**Published:** 2024-01-02

**Authors:** Nadlada Tawankanjanachot, Maria Truesdale, Pornpun Orachon, Lisa Kidd

**Affiliations:** 1 School of Medicine, Dentistry & Nursing, College of Medical, Veterinary & Life Sciences, University of Glasgow, 57-61 Oakfield Avenue, Glasgow, G12 9LL UK; 2 School of Health and Wellbeing, College of Medical, Veterinary & Life Sciences, University of Glasgow, Glasgow, G12 0XH UK; 3 Yuwaprasart Waithayopathum Child and Adolescent Psychiatric Hospital, Department of Mental Health, Ministry of Public Health, Samut Prakan, 10270 Thailand; 4 Ramathibodi School of Nursing, Faculty of Medicine Ramathibodi Hospital, Mahidol University, Bangkok, Thailand; 5 School of Health & Life Sciences, Research Centre for Health (ReaCH), Glasgow Caledonian University, Glasgow, G4 OBA UK

**Keywords:** Social skills intervention, Adolescents with autism spectrum disorder, Qualitative research, Experiences, Caregivers, Healthcare professionals

## Abstract

**Background:**

Social skills interventions (SSIs) are effective for enhancing social skills and decreasing mental health problems in adolescents with autism spectrum disorder (ASD). However, these interventions have been designed and their effectiveness has been established in Western countries. Lack of culturally acceptable SSIs for Asian nations is a possible barrier to implementing effective and tailored interventions that address the unique requirements of ASD individuals across countries and cultures. This study aims to explore the needs and preferences of adolescents with ASD, their caregivers, and healthcare professionals (HPs) in Thailand regarding the components, delivery formats, and cultural adaptation required for an outpatient-based social skills intervention.

**Methods:**

Qualitative data was collected via three focus groups of HPs (n = 20) and 24 paired interviews with adolescents with ASD and their caregivers from a child psychiatric hospital in Thailand. Purposive sampling was employed, and thematic analysis was used to analyse the data.

**Results:**

Nine themes emerged from the data generated by HPs, and seven from adolescents with ASD and their caregivers. SSIs for Thai adolescents with ASD and their caregivers should emphasise specific social skills training and assess the abilities of adolescents as required. Incorporating various learning strategies is important. Parental involvement is essential and provides knowledge of an adolescent’s symptoms and coaching skills, which are best used to support their adolescents. Cultural considerations include the need for social knowledge of Thai culture, promoting assertiveness and praising parents’ abilities, implementing a programme in time to not interrupt academic achievement, and renaming a programme from social skills intervention to social communication intervention. Barriers to implementing a programme included HPs’ need for specialised training and education and decreased workload. Also, the caregivers’ and adolescents’ stigma reduced attendance in a programme. Increased extra compensation and relocation days off are provided as policy support for staff who deliver the intervention.

**Conclusion:**

The results suggest that SSIs for Thai adolescents with ASD should be tailored to meet the needs for specific knowledge, skills, and parental collaboration as coaches for their adolescents. Additionally, it should incorporate Thai culture. It is necessary to consider staff knowledge, workload, and stigma in order to reduce barriers to implementation in practice.

**Supplementary Information:**

The online version contains supplementary material available at 10.1186/s13033-023-00617-3.

## Background

### Evidence and current understanding of social skills interventions for adolescents with ASD

Social skills impairments are core problems experienced by adolescents with autism spectrum disorder (ASD) [1] and have an effect on their development in the long term [2]. This includes difficulties with social-emotional reciprocity, social interaction, and forming and maintaining relationships with others [3]. Social skills interventions (SSIs) are interventions that help individuals with ASD to learn social norms (e.g., conversation skills, play skills, and nonverbal communication) [4] and are predicated on the concept that difficulties in social interaction are the result of an insufficiently developed understanding of pertinent social rules [5]. Interventions for social skills typically include multiple components such as behavioural therapy, parent training, and peer modelling [6, 7] and are delivered in a variety of formats such as individual or group [8]. SSIs may increase the child’s awareness of social cues and teach strategies for social problem-solving [9]. Effectiveness of SSIs in improving social skills and reducing mental health problems in adolescents with ASD has also been demonstrated [10, 11]. In particular, previous studies, conducted in Europe and America, have shown that group-based SSIs can play a significant role in improving social competence [4, 12], quality of life and emotion recognition [13] in adolescents with ASD. In addition, studies have shown that SSIs can be useful in reducing social anxiety amongst adolescents [14, 15].

Evidence of the effectiveness of SSIs for adolescents with ASD in non-Western countries, however, is more limited. A recent systematic review of SSIs for adolescents with ASD in Asia, conducted by the author [16], highlighted that although some interventions have been found to be effective, there is a need for further research to explore how interventions align with the cultural norms and expectations of people living in non-Western countries and their subsequent effectiveness in improving outcomes such as social competence [4, 12], quality of life and emotion regulation [13] The review also used the Ecological Validity Model (EVM) [17], which provides a structure for understanding what can be adapted in the delivery and content of an intervention (details shown in Table 1). It served as a framework to investigate the extent to which existing SSIs have been culturally adapted for adolescents with ASD in Asian countries. The lack of culturally appropriate SSIs for use in Asian countries is a potential barrier to the delivery of appropriate and tailored interventions that address the diversity of needs of individuals with ASD in different countries and cultures [18] and thus this is an important area to address through future research.

**Table 1:** The components of the EVM framework [32]

Components	Definitions
**1. Language**	The process of linguistic adaptation, translation and appropriate culturally syntonic language.
**2. Persons**	The influence of the culture group’s characteristics on therapy relationships between therapist and clients.
**3. Metaphors**	Symbols and images shared with the population’s culture which make them feel comfortable. Using language metaphors to reduce resistance and increase motivation.
**4. Content**	Cultural knowledge of values and traditions influence the therapeutic goal.
**5. Concepts**	Treatment concepts consonant with culture and context: dependences vs. independences
**6. Goals**	Treatment goals are framed in keeping with the culture’s values, customs, and traditions.
**7. Methods**	Methods or procedures for accomplishing objectives are aligned with the culture.
**8. Context**	The social, economic, and political environment and how changes in these domains may affect the patient.

### Care for adolescents with ASD in Thailand

Thailand, a country in Southeast Asia, has a growing prevalence of children and adolescents with ASD but limited evidence of the needs for and effectiveness of SSIs. The prevalence of ASD in Thai children under the age of 12 years has increased dramatically from 1.43 per 10,000 in 1998 to 6.94 per 10,000 in 2002 [19]. In 2021, there were 19,106 Thai adolescents with ASD aged between 10 and 19 who received healthcare for ASD, compared with 7.8 million adolescents in Thailand overall (245 per 100,000) [20]. According to recent estimates, just 15% of 370,000 people with ASD in Thailand, have access to ASD-specific healthcare services [21]. Health service provision from the Thai government begins with early detection in screening children for ASD and early intervention in general hospitals between the ages of 0–6 years [22, 23]. Tertiary care is also provided for children and adolescents with ASD through specialist psychiatric hospitals [24], however, anecdotal evidence suggests that few SSIs programmes are currently offered through tertiary care centres for adolescents with ASD and their families. One group-based programme that does exist in the Yuwaprasart Waithayopathum Child Psychiatric Hospital, located in Central Thailand, was established to provide inpatient intensive support for adolescents with ASD and their families [25]. However, this has not been rigorously evaluated. Furthermore, there have been no previous attempts to understand the requirements for adaptation of a social skills programme for an outpatient-based setting, which would help to support the transition of adolescents and their families across acute and community-based care [26, 27]. It is widely acknowledged that change and transitions of care might be challenging for this population [28, 29].

There have not been any intervention studies published in Thailand to date, and no qualitative studies which have explored the experiences of ASD in Thailand from adolescents’ and families’ perspectives. Therefore, in the absence of existing evidence that addresses the specific cultural dimensions of Thai adolescents and families in relation to social skills interventions, there is a need to explore what modifications and cultural adaptations are needed to ensure that such interventions are suitable and appropriate to the needs of adolescents with ASD in Thailand [30].

In order to determine the needs of, and preferences for, the components, formats of delivery and cultural adaptation required for an outpatient-based SSI for adolescents with ASD in Thailand and their families, further qualitative research is needed. Thus, to address these gaps, the current study was undertaken using qualitative methods to identify the cultural issues and social expectations of SSIs from different stakeholder perspectives. The study also identified the barriers and facilitators to the delivery and implementation of SSI in an outpatient setting in Thailand.

## Research questions

1. What are adolescents with ASD’s, families’ and healthcare professionals’ experiences and perceptions of social skills problems and their management in Thailand?
2. What are the cultural issues and social expectations in Thai culture that could impact the content and delivery of an outpatient-based SSI for adolescents with ASD?
3. What are the perceived barriers and facilitators to implementing an outpatient-based SSI in Thailand?

## Method

### Design

This qualitative study represents the second phase of a programme of work conducted by the authors guided by Barrera and Castro’s heuristic cultural adaptation framework [31] and Bernal’s Ecological Validity Model (EVM) [32] to determine the culturally appropriate content, structure, and formats for delivery of an outpatient based SSI for adolescents with ASD in Thailand. Barrera and Castro (2006) suggest the steps for culturally adapting an intervention in four phases: (1) gathering of information (2) preliminary adaptation design, (3) preliminary adaptation tests, and (4) adaptation/refinement [31]. The work described in the aforementioned systematic review [16] and the current qualitative study described here align with the first phase of Barrera and Castro’s (2006) framework (gathering of information). In addition, the EVM framework was used to analyse cultural components in relation to SSIs fitting the needs of Thai adolescents with ASD. There are eight components which are described in Table 1 [17].

### Qualitative method

Building on the systematic review findings, the current study adopted a qualitative research methodology to understand the needs, expectations, and experiences [33] of social skills problems and interventions for adolescents with ASD from the perspectives of Thai adolescents with ASD (aged 10–19 years), their caregivers, and healthcare professionals (HP/HPs) [34]. This qualitative study used two types of qualitative data collection, including: (i) focus groups with HPs working with adolescents with ASD, and (ii) paired interviews with adolescents with ASD and their families/caregivers. Focus groups were employed to encourage a space where HP participants could comment, explain, agree or disagree with each other, and share opinions amongst each other that might not surface during individual interviews. Paired interviews were employed with adolescents and their caregivers because of the sensitivities around, and complexities that adolescents with ASD may face, in communicating with other people and because the presence of a family member can often support this [35, 36]. Furthermore, the paired interview approach also allowed for a dialogue to happen between the researcher and the adolescents or caregivers, as well as between the adolescents and the caregivers, which was helpful for further context and explanation, and for comparison, cross-checking, and triangulation [37] between the information given by both adolescents and their care givers. All the focus groups and interviews were conducted in the Thai language.

### Study setting

The study was conducted at Yuwaprasart Waithayopathum Child Psychiatric Hospital in Thailand between April and July 2021. This public tertiary hospital in Thailand has the largest number of cases of individuals with ASD among mental health hospitals in the country [21].

### Sampling and sample size

There were three groups of participants, including adolescents with ASD, their family caregivers, and HPs. The study used a purposive sampling technique, informed by a sampling framework (Tables 2 and 3), to ensure heterogeneity across all groups of participants and to capture as diverse a set of experiences and perceptions as possible [38, 39]. The sampling frame for HPs was grounded in the context of the current staff mix at the hospital and was used to ensure representation from different professional groups within the HPs sample that would have experience or an understanding of SSIs for adolescents with ASD (Table 2). The author’s previous systematic review identified that HPs (all career in Table 2) were the key people involved in the delivery of SSIs for adolescents with ASD [16]. Consequently, these staff have experience of implementing SSIs and can give information about what SSIs will look like and any issues relevant to Thai culture that they have experienced previously that might influence the acceptability or effectiveness of these for adolescents with ASD in Thailand [40, 41]. The target sample size for HP participants was 18 which was believed to be achievable within the available pool of staff and would allow sufficient depth to the qualitative analysis. The inclusion criteria for HPs were multidisciplinary staff (e.g., psychiatrists, nurses, psychologists, special education, social workers, occupational therapists, and speech therapists) who worked in the selected hospital and provided care for adolescents with ASD whether in the inpatient or outpatient clinic.

**Table 2:** Sample frame of HPs

Discipline/ professional background	Number of staff potentially available to recruit	IPD (SSI ward)	OPD	Work in IPD and OPD	Target sample number	Final participants
Child Psychiatry	9	-	-	9	3	3
Psychologist	6	-	-	6	3	3
Nurse	40	32	8	-	7	6
Special educator	5	-	-	5	2	2
Speech therapist	2	-	-	2	1	2
Social worker	4	-	-	4	2	3
Occupational therapist	-	-	-	-	-	1
**Total**					**18**	**20**

*IPD – inpatient department, SSI – Social skills intervention, OPD – outpatient department

For the adolescents with ASD and their caregivers, characteristics such as age, gender, and previous experience with or participation in SSIs were considered important within this study and framed the recruitment strategy (Table 3). These characteristics were important to consider in this study because they are known to affect engagement with and the experiences and outcomes of SSIs [42–46]. This study also included both participants who had experience of SSIs and individuals who had never been part of a SSI, as the experiences of both groups were believed to be potentially different and their perspectives useful for highlighting the key ingredients and factors of an intervention influencing their initial or sustained engagement. These insights were considered to be important for understanding the acceptability and effectiveness of SSIs [47]. The target sample size for adolescents and caregivers was 32 (16 in each group) which again, was generated from an estimate of the available pool of adolescents and caregivers and would allow sufficient depth to the exploration of their needs and experiences. The inclusion criteria for adolescents and their caregivers were adolescents aged between 10 and 19 years with a diagnosis of ASD (by DSM-IV, V, or ICD-10) and who had level of IQ more than 70. Caregivers were eligible if they were informally caring for or a parent of an adolescent with ASD (who met the above criteria).

**Table 3:** Sample frame of adolescents with ASD and their caregivers

Characteristic	Detail	Number of patients at the hospital	Estimated number of participants
**Age** (10–19 years)		2020	
• 10–14	Early stage	3,075	3–4 person
• 15–17	Middle stage	1,290	3–4 person
• 18–19	Late stage	519	3–4 person
**Gender**	Prevalence of ASD male: female 4:1	-	
Male	4		12 males
Female	1		4 females
**Experience with social skills intervention**			
**-** Adolescents who have been part the social skills intervention	-Adolescents with ASD who had ever attended a social camp at the in-patient clinic between 2017 and 2019	101	8
**-** Adolescents who have never participated in a social skills intervention	- Adolescents with ASD who never attended the social camp		8

A total of 20 HPs from the hospital were recruited, as well as 24 adolescents with ASD and 24 caregivers. Data saturation occurred during the 22^nd^ paired interview and after three focus groups, when all participants met the inclusion criteria, and the codebook included no new information [48–50]. To ensure that no additional information was gained, the researcher opted to interview two additional caregivers and their adolescents [51].

### Recruitment and consent

To identify participants, the main researcher (NT) worked with a psychologist team leader (PO) from the hospital’s behaviour clinic who was also involved in making the initial approach to and recruiting participants for the study. Recruitment approaches used included an open advert via a study poster in the hospital and identifying potentially eligible adolescents from the clinic list who met the inclusion criteria. Caregivers were identified by the adolescent themselves or by those family members who are also listed in the adolescent’s clinic records. The HPs were identified by the psychologist team leader. If they met the inclusion criteria, they were sent an invitation letter distributed by PN to consider participating. They had the autonomy to provide informed consent and could withdraw from the research without any negative consequence [52, 53]. The assent form (for adolescents with ASD) was adapted from a study by Dockett, Perry (2013) which used pictures and simple language [54]. The researcher provided an assent form because the adolescents had the autonomy to provide informed consent [55–57]. All of the participants signed a consent form or an assent form and gave permission to record the audio of the interview.

### Data collection

The topic guides for the paired interviews and focus groups were developed from the findings of the systematic review (Additional file 1and 2) and were translated into Thai language prior to use. The paired interviews guide focused on caregivers’ and adolescents’ experiences and expectations for the kinds of support that would be beneficial for social skills development. Additionally, the focus group guide discussed HP’s expectations for SSIs and how this could be accomplished in an outpatient hospital setting.

The focus group topic guide was tested prior to use with two HPs in the hospital. The paired interview topic guide was tested with two adolescents with ASD and their caregivers to ensure that all questions were clearly understood by participants [58]. Following the pilot test, only minor changes were made to both interview guides. The data from the pilot focus groups was not included in the study analysis (because the participants did not fully meet the inclusion criteria) but the interview data from the pilot of the paired interviews with adolescents and caregivers was included in the study analysis.

Three focus groups of HPs (n = 6, n = 6, n = 8) were conducted in a quiet meeting room in the hospital and lasted from 1 ½ to 2 ½ hours. Demographic data of HPs was collected on age, gender, level of education, and information on experience of working with individuals with ASD. Paired interviews (n = 24) of adolescents with ASD and their caregivers were conducted in a quiet room in the hospital at the time of their convenience and lasted between 47 mins − 2hrs and 51 mins. All participants completed the demographic questionnaires (e.g., age, gender, the caregiver’s highest level of education, and information about the adolescent’ s autism diagnosis and its management) before the interviews.

### Ethical considerations

Research ethics approval was given by the University of Glasgow (number: 200200047) College of Medical, Veterinary and Life Sciences (MVLS) Research Ethics Committee on February 10, 2021, and by the institutional review board of Yuwaprasart Withayopathum child and adolescent hospital committee (number 07/2021) on April 1, 2021. Since the adolescents in the study were considered a vulnerable group, a number of additional measures were taken, including a protocol for managing any emotional distress during the interviews [59] and an information sheet with pictures and simple language to aid communication during the study [60, 61]. Participants were reminded that their participation in the study was voluntary and that their data would remain confidential. Audio recordings were transferred into an encrypted computer and once transcribed were deleted.

### Data analysis

After the completion of each interview and focus group, the audiotapes were listened to carefully and then transcribed verbatim into MS Word documents in Thai, the main researcher’s mother tongue. The translation technique reported by Chen and Boore (2010) was used to identify patterns or themes in the same language as the interview and then translate only the concepts, categories, and themes into the English language [62]. Interview transcripts were analysed using thematic analysis [63]. This technique is used to identify, analyse, organise, describe, and report themes found in qualitative data [64, 65]. The approach outlined by Braun and Clarke (2006) was adopted in the current study as follows: (1) familiarisation with the data, which involved transcribing the interviews verbatim in the Thai language and reading the transcripts and field notes from each interview, (2) data coding, where initial codes were identified and applied across all of the transcripts (3) searching for themes, where codes were gathered and grouped into thematic categories. (4) reviewing themes, which involved reviewing the themes across all transcripts, revising these exemplar quotations into English and, (5) defining and naming the themes [64]. This process was done independently and concurrently by the researcher (NT) and supervisors (MT, LK). Data saturation was obtained when there was no new theme or information [48, 66].

## Results

Our findings are presented in two parts: demographic characteristics of the study participants, and the main findings comprised of emerging themes from the data.

### Participant demographics

The participants in this study included 20 HPs, 24 adolescents with ASD and 24 caregivers. Table 4 outlines the characteristics of the HPs. In total, 20 of the participants were female and two were male. The sample included nurses (n = 6), child psychiatrists (n = 3), psychologists (n = 3), social workers (n = 3), speech therapists (n = 2), special educators (n = 2), and occupational therapists (n = 1). Seven of those who took part had more than 20 years’ experience in caring for people with ASD. The majority (n = 15) worked in both the inpatient and outpatient departments.

**Table 4:** Characteristics of HPs

	HPs (n = 20)
**Gender**	
Female	18(90%)
Male	2 (10%)
**Age**	
21–30	5(25%)
31–40	5(25%)
41–50	9(45%)
51–60	1(5%)
**Education**	
Bachelor’s degree	13 (65%)
Master’s degree	7 (35%)
**Function**	
Child psychiatrist	3 (15%)
nurse	6 (30%)
psychologist	3 (15%)
social worker	3 (15%)
speech therapist	2 (10%)
special educator	2 (10%)
occupational therapist	1 (5%)
**Years of work with ASD**	
2–5 years	6 (30%)
5–10 years	1 (5%)
10–15 years	1 (5%)
10–19 years	5 (20%)
> 20 years	7 (35%)
**Type of department**	
OPD	2 (10%)
IPD	3 (15%)
OPD and IPD	15 (75%)

Table 5 summarises the characteristics of the adolescents and caregiver participants. In total, 12 were considered to be in early adolescenece, (10–14 years), nine in middle adolescence (15–17 years), and three in late adolescence (18–19 years). Fifteen adolescents attended secondary school, five attended primary school, three attended universities, and one attended a vocational diploma programme. Thirteen of twenty-four adolescents were diagnosed with ASD, three with Pervasive Developmental Disorder-Not Otherwise Specified (PDD-NOS), two with Asperger’s syndrome, four with combined ASD and Attention Deficit Hyperactivity Disorder (ADHD), and two with comorbid ASD and depression. Half of the adolescents had nearly normal cognitive abilities, and the other half had average (90–109) to above-average cognitive abilities (110–119) (as measured by WISC-IV). In total, 13 adolescents [13] had previously participated in a social skills intervention programme. The caregivers included 22 females and two males. More than half of the caregivers had a bachelor’s or master’s degree.

**Table 5:** Characteristics of adolescents with ASD and their caregivers

	Adolescents with ASD (n = 24)	Caregivers (n = 24)
**Gender**		
Female	5 (20.83%)	22 (91.67%)
Male	19 (79.17%)	2 (8.33%)
**Age**		
10–14	12 (50%)	
15–17	9(37.5%)	
18–19	3(12.5%)	
31–40		7 (29.17%)
41–50		10 (41.67%)
51–60		7 (29.17%)
**Education**		
Primary school	5 (20.83%)	1 (4.17%)
Secondary school	15 (62.50%)	7 (29.17%)
Vocational Diploma	1 (4.17%)	3 (12.50%)
university	3 (12.50%)	
Bachelor’s degree		8 (33.33%)
Master’s degree		5 (20.83%)
**Diagnosis**		
ASD	13(54.15%)	
PDD-Nos	3(12.5%)	
Asperger	2(8.33%)	
ASD with ADHD	4(16.67%)	
ASD with Dysthymia or Depression	2(8.33%)	
**Level of IQ**		
70–79	5 (20.83%)	
80–89	7 (29.17%)	
90–110	9 (37.50%)	
111–120	3 (12.50%)	
**Previous experience with social skills training interventions**		
No	11 (45.83%)	
Yes	13(54.17%)	

**Fig. 1:**
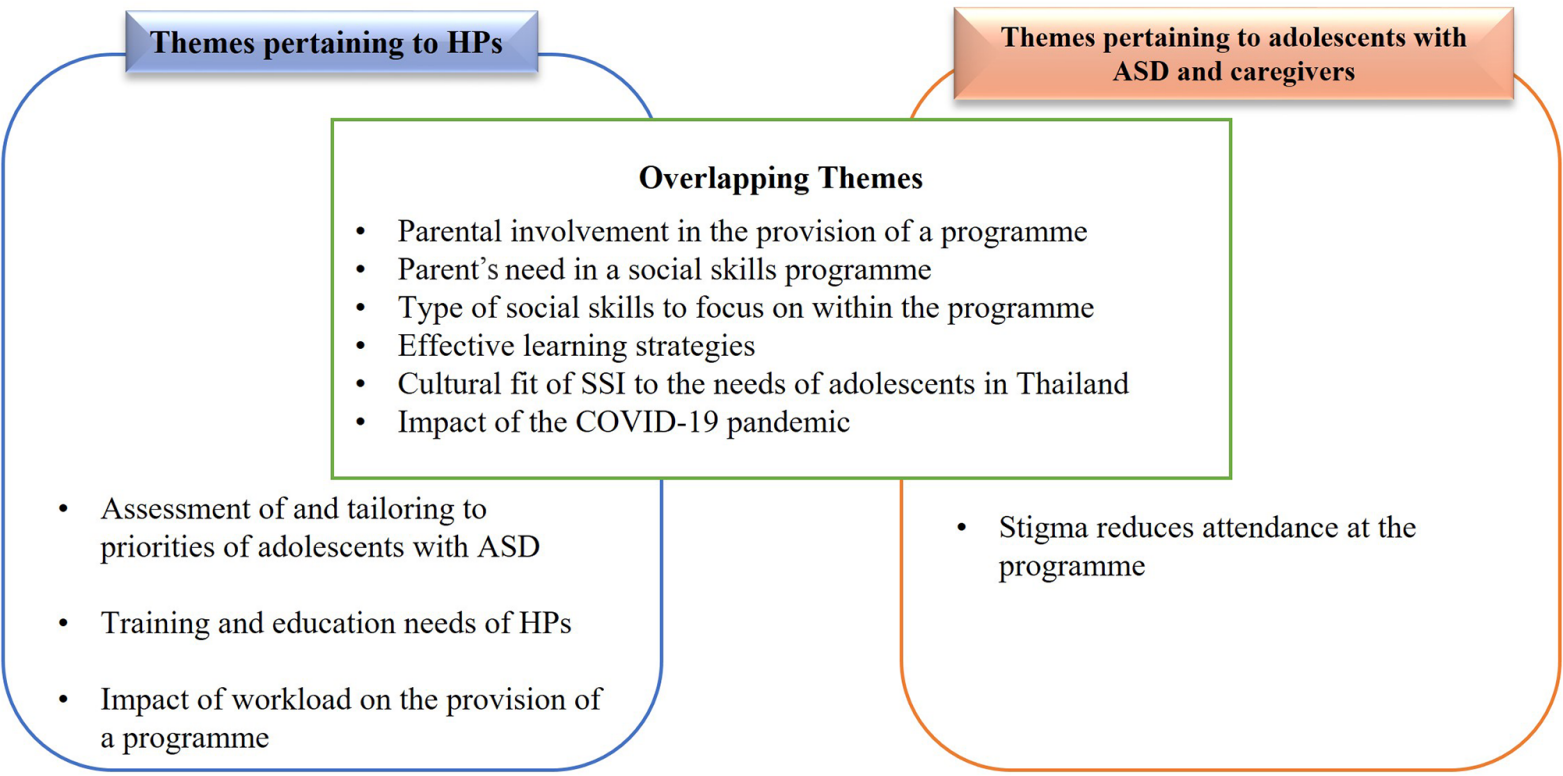
Themes of HPs and adolescents with ASD and their caregivers

Figure 1 depicts the themes that were constructed during the analysis. Given the overlap that existed between several themes, the following sections firstly present the themes that pertained to both the HPs and the adolescents, and their caregivers (Sect. 1) followed by the unique themes identified for each group separately (Sect. 2 and 3). In each section, examples from the focus group and interview data are included to show evidence of the theme and its interpretation.

### Themes pertaining to both of HPs and adolescents with ASD and their caregivers

The following section presents the themes from the perspectives of both HPs and adolescents with ASD and their caregivers. In the exemplar quotes, HPs are denoted by the participant’s disciplinary background, their study number and the focus group number that they participated in. Adolescents’ quotes are denoted by the adolescent’s gender, age and experience of social skills programme. Caregivers’ quotes are denoted by the word caregivers and then information pertaining to the adolescents they care for is given. Four main themes were constructed from both groups including (i) Parental involvement in the provision of a programme (ii) Parents’s needs in a social skills programme.

(iii) Types of social skills to focus on within a programme (iv) Effective learning strategies (v) Cultural fit of SSIs to the needs of adolescents in Thailand (vi) Impact of the Covid-19 pandemic.

#### Parental involvement in the provision of a programme

This theme was constructed from the discussions that both groups had around parents’ engagement in the provision of a social skills programme for adolescents with ASD. Parents have a significant role and influence in the development of social skills in adolescents with ASD. HPs believed that if a programme can encourage parental participation as a co-therapist during treatment, adolescents with ASD will receive continuous treatment and significantly improve their social skills.

I see the training gap in our programme. We have been training [people with] ASD since they were young, but we never invited the parents to the training session. We always suggest to the parents that they should follow the exercise and keep training their child, but they don’t know how. They don’t know the training procedure and technique. Their kid, then, was stuck at that point, no further. (Nurse 05: FG3)

The most important thing is parent cooperation. As a multidisciplinary team, we cannot take care of and train the autism children for the whole time. We need parental cooperation. The skill training absolutely affects parents’ daily routine, but it is not sustainable. I think that we just started some social skills and parents should keep him exercising those skills frequently around his situation. If parents ignore this exercise or their role, the child’s skills won’t improve. (Social worker 03: FG3)

This is similarly true for adolescents with ASD and their caregivers who perceived it was important for them to be engaged in a programme. Parents believed that if they too received the training in a programme, they could enhance their adolescent’s development and help to resolve social skills problems in daily life. In addition, many adolescents rely on their parents’ competence in order to care for and coach them when they encounter difficulties, so it was important that they knew how to appropriately and effectively support their adolescents.

That is necessary if I am to attend a programme. At least, I would like to understand what they [HPs] teach my son, what parents should do after that, and how parents handle them. Some parents don’t understand a child; they use general behaviour to control a child. However, if I understand what he thinks, or he thinks only that, I then understand his intention and what he thinks. The parents’ expectations are reduced. We understand each other. There is no pressure on children too. (Caregiver of male aged 18 years, previously attended SSI)

I think my dad could join in the programme. He can teach me well. He can warn or teach me what and how to do that properly. If I meet a new person, I don’t know anything, but if there are parents guiding me on how to communicate, that helps. (Male aged 13 years, not attended SSI)

#### Parent’s needs in a social skills programme

This theme was constructed from the discussions that both groups had around the knowledge and skills that are important for parents. The HPs mentioned that parents need knowledge of their child’s symptoms, how to intervene to help address social skills problems, and coaching with the opportunity to practice skills development. Parents also identified a need for education and the opportunity to practice skills development, as well as individual counselling for example in how to manage family issues and conflict. HPs mentioned that most parents have high expectations for, and prioritise, the academic learning abilities of their adolescents rather than focusing on improving their social skills because they lack knowledge about how important treatment is. Furthermore, the existing social skills intervention within the hospital was primarily focused on social behaviour issues. Therefore, parents may believe that their adolescents will recover once the problem is resolved.

Parents have certain expectations, such as wanting their child to speak and behave well at school. They understand that being in school promotes socialisation. Another expectation is that the child can attend the classroom. However, once their child can go to school, it doesn’t mean that their social skills problems are solved. (Psychologist 01: FG1)

To address these problems, HPs believed they could collaborate with parents to establish treatment goals and impart knowledge, including (i) ASD and their social skills problems, how to address them, (ii) adolescent development, (iii) coaching skills, and (iv) treatment goals and objectives. These knowledge and skills were perceived to have an effect on the caregiver’s perception of the illness of their child.

Actually, I think that parents should know why we teach that subject and why that subject is important. The subject could start with some conventional social skills problems. Parents would immediately recognise that their child is related to each of them. I prefer parents to get the basic knowledge of social skills problems in an autistic child. It may not be relevant to all areas, but there should be at least one problem. (Child psychiatrist 01: FG1)

Parents also identified a need for knowledge about ASD symptoms, social skills deficits in adolescents with ASD, strategies for improving social skills and communicating with their adolescents, as well as strategies for enhancing their adolescent’s development. They perceived that this kind of education and training could help them to understand and know how to support their adolescents and help them to reinforce the learning achieved through a programme whilst at home.

That is necessary if I am to attend a programme. At least, I would like to understand what they teach my child, what parents should do after that, and how parents handle them. Some parents don’t understand a child; they use general behaviour to control a child. However, if I understand what he thinks, or he thinks only that, I then understand his intention and what he thinks. The parents’ expectations are reduced. We understand each other. There is no pressure on children too. (Caregiver of male aged 18 years, previously attended SSI)

In addition, the majority of HPs reported that many parents feel additional stress when they are caring for their adolescents with ASD. Thus, group support (e.g., counselling, parent-parent peer group) could be beneficial within a social skills programme.

Some parents have mental health problems, because their child has had a lot of problems since they were born. Parents have to confront this issue every day for many years. These parents really need some mental health support. They might not see improvement in their child, but if they acknowledge the issue and we support them, they will. They continue to walk and look after their kids. (Special educator 02: FG2)

#### Type of social skills to focus on within the programme

This theme was developed based on both groups’ experiences and consequences of social skills impairments, as well as the identification of the areas of social skills that adolescents with ASD need to strengthen. According to the experiences of both groups, they found that the adolescents faced problems when they had interaction with other people because of deficits in social skills. They also believed that social skills training could assist individuals in socialising, understanding, and coexisting happily with other people. Both groups agreed that the social skills that adolescents need to strengthen are in three areas. 1) Social communication: the adolescents and their caregivers thought that this skill is necessary for developing and maintaining relationships with others (for example, back and forth conversation, sharing their interests, assertiveness, how to interact with their friends, how to develop the relationship, and how to make new friends).

I don’t know what subject I should talk about with them. We can chat [together] for a while, then separate. I feel weird. Yeah, it’s err. quiet atmosphere. I don’t know what subject I should continue after that. (Female aged 18 years, previously attended SSI)

Similarly, the HPs mentioned, this skill is predominantly pragmatic (refers to the social language abilities that we use in our everyday interactions with others). One of the HPs identified the importance of this skill. Another HP stated that they never teach these particular skills, which are the ones that adolescents with ASD struggle with the most.

We call it a pragmatic skill. For example, when a group of children is talking or discussing, a child should be able to read his friends’ facial expressions, understanding when they don’t want to talk anymore. A child should also be capable of waiting and listening to his friends’ discussions. A child won’t talk only about the topics he is interested in; he should know that what he says affects his friends’ feelings. He needs to be aware of what he should say and what he shouldn’t. He should understand when to maintain the topic or take turns. These are pragmatic skills that a child should learn first. However, I acknowledge that it is challenging to train these skills because I work with children in individual sessions. Pragmatic skills should be trained in group sessions, with other friends. (Speech therapist 02: FG2)

2) Theory of Mind (TOM) is the second social skill that both groups mentioned. This skill is essential for understanding how other people think and behave when interacting.

I think TOM should be trained early. Lacking TOM leads to a lot of problems. Some adolescents with ASD have emotional control, but they don’t have TOM. They would misinterpret and take action immediately. For example, they kick, punch, or blame their teachers when their teacher teaches them. (Child psychiatrist 01: FG1)

CG 15: Sometimes, he is too sensitive. For example, when his grandmother comes back from work and she feels tired, she just goes to sleep with her flat facial expression. He then interprets if his grandmother feels stressed or if she is sick. He observes other people’s facial expressions and always interprets that one may be stressed or angry. He always thinks that he is the cause of those expressions. (Caregiver of male aged 15 years, previously attended SSI)

3) The last category of social skills that both groups mentioned were difference types of social skills outcome. The HPs mentioned problem-solving skill, while the caregivers and their adolescents thought about emotional regulation, which is a constant challenged when their peers tease or bully them.

When he cannot express himself, it does affect his emotions. (Caregiver of male aged 11 years, not attended SSI)

My friend teased me about Dodo. He teased me a lot. I just couldn’t stand it. That time, my mom was not at home. I ran directly to him, pulled him down, and punched him, but I missed. That friend was surprised. (Male aged 10 years, not attended SSI)

#### Effective learning strategies

This theme was developed based on both groups’ experiences with the most effective learning strategies for improving, monitoring clinical outcomes, and increasing group training participation amongst adolescents with ASD. As learning strategies, both groups referred to two key strategies (homework and assignments), as well as peer modelling. In addition, the HPs mentioned multimedia, and the caregivers and adolescents mentioned the activities in the programme and group sessions.

In relation to homework and assignments, these were encouraged for both parents and adolescents with ASD to gain a better understanding of the elements of the training programme itself. Many adolescents’ homework assignments require online resources, which can be practised after the session. These resources could inform them on how to improve their social skills, such as through the use of websites or video clips online. These materials can help them practise by themselves.

Homework assignments are essential because we cannot simply assign verbal or timetable assignments. We would make an agreement with parents, finalise it into several steps, determine what they should do, how frequently it is, and what or how they assess. Thus, the homework assignment is very important, related to our training objective and flexible to their family context. (Psychologist 01: FG1)

I need homework and the knowledge information that I want on the computer [on the website], but on the actual paper, there is less information. (Male aged 13 years, not attended SSI)

The homework assignment fulfils the missing skill. If we have that, I could act with her at home. For the difficulty level, I have to check it first. If I act with her, I can do that. There is no problem for me. (Caregiver of female aged 10 years, previously attended SSI)

Peer-modelling refers to teaching strategies used by typically developing children to facilitate social interactions with ASD adolescents, which is beneficial for gaining real-world experience with social skills. Additionally, the parents and their adolescents perceive that ‘typically developing” adolescents who prefer to volunteer serve as positive role models and can motivate those with ASD to participate in activities.

If these normal children have been training with us, they might be some good teacher assistants. When they have a good attitude, they earn self-esteem. They could help others. Then, the autistic child could imitate good behaviour. This could be done during school break. (Psychologist 01: FG1)

There should be some normal children because he would understand how a normal child act. It could give him some examples and they can talk with each other. If the parents are in the same session, that would be good because I would know how other people thinks. Each child has a different style, I can see and understand them. (Caregiver of male aged 15 years, not attended SSI)

HPs also mentioned multimedia (such as video clips) in relation to supporting communication and how adolescents with ASD may benefit from the use of multimedia with video clips with animation, sound, and social content relating to their deficit in social skills. This type of media was perceived as being of higher quality and more interactive than pictures and more attractive to adolescents. They are also advantageous for teaching when participants are unable to attend a hospital session due to the fact that they retain more information after viewing multimedia.

I think that the current social skills training material is of poor quality. It doesn’t clearly help autistic adolescents understand. If we had various useful training materials of good quality, it could help us make parents understand clearly. For instance, having video clips would allow us to demonstrate proper behaviours for the adolescents to observe. These clips should be categorised into different daily situations. Currently, the existing media in Thailand mainly consists of books. I would prefer some useful video clips for parents and autistic adolescents. This media could assist us in guiding them during individual or group sessions. (Special educator 01: FG1)

The activities in a programme and group sessions were mentioned only by the adolescents with ASD and their caregivers. They mentioned that the training programme could provide more activities that are fun and interesting (e.g., playing online, sports), and some activities should be carried out in a real-world setting where they can practise the skills (e.g., museum, theatre). These activities are enjoyable and encourage adolescents to develop relationships with others.

Apply the game, make a game to do some activities in the class, or bring those famous games into the classroom to make children feel interested and concentrated. If one could answer correctly, he would get a reward, etc. (Male aged 15 years, previously attended SSI)

In my view, the staff may ask children what they like first, e.g., cartoons or science. Then, staff group them together. If they have the same topic that they like, they will have several ideas and discussions. Emm. The staff provides the neutral activity. However, my daughter says she wants something funnier than that. She thinks that it is not interesting, but the staff let her work with friends. It seemed like she was forced to do it. (Caregiver of females aged 18 years, previously attended SSI)

Additionally, caregivers and their adolescents mentioned that a social skills programme could provide more group sessions in which the adolescents could practise the skills they had been taught in a group setting. It was reported that ideally participants in this training group should be of similar ages, abilities, and interests. Due to the ease with which similar experiences can be shared, this can motivate them to participate in group activities.

The age of the group members should be taken into account. Mostly, they [HPs] should set the programming according to age, so they stay together. They would understand each other if they worked on a group project. They should be the same age. Different ages have different issues. It should be separated. Their ages should be close, e.g., pre-teen age. (Caregiver of male aged 15 years, previously attended SSI)

#### Cultural fit of SSI to the needs of adolescents in Thailand

This theme was analysed based on the needs of Thai culture in relation to social skills intervention from the perspective of both groups and aligned to relevant components of EVM model [17, 32]. Both groups discussed the person, content, and method components. While the components of goal and metaphor were mentioned, only HPs commented. These components can increase the engagement in the training programme and tailor it to fit the culture.

##### Person:

The value of attending school over participation in the programme affected therapeutic relationships and programme participation. Regularly attending the training is important. However, many parents believed that school provided their adolescents with adequate socialisation and academic learning. Their adolescents were allowed to attend school if they were able to communicate and exhibited no disruptive behaviour. The caregivers and their adolescents mentioned that training should happen on weekends or when school is out of session. This time might facilitate treatment collaboration.

Parents have high expectations in academic learning. As I’ve observed, if a child can go to school, learning skills are more important than behaviour training. (Psychologist1: FG1)

I talked to my patient. His grade has improved from 1.7/4 to 2.4/4. The parents think that their kid is doing better, so they skip or postpone the training. I have to ask the parents if they want to continue training because the schedule is disrupted. But the patient asks me, ‘If you say that I need to practice social skills, how come I take a leave from school? (Child psychiatrist1: FG1)

I prefer group training on weekends because he studies so hard on weekdays. He has never stopped learning. During grades 7–9, he tried to go to school even when he was sick. (Caregiver of male aged 15 years, previously attended SSI)

##### Metaphor:

Renaming the programme can make parents feel comfortable and concerned about participating in the programme. HPs mentioned that the term “social skills intervention” could be changed to “social communication intervention” because parents are more concerned with communication than social skills.

If we [HPs] name the programme “social skills programme”, it becomes quite interesting. Furthermore, it could be a social and communication skills training programme. Communication is a problem they face. Most parents are concerned about social skills at the end of the cause, but they prioritise communication as the top concern. (Psychologist1: FG1)

##### Content:

The cultural knowledge of Thai tradition that both groups mentioned, which is impacting on teaching social skills was included.

The HP mentioned a varied hierarchical structure based on an individual’s time, place, and setting; morality; obedience; generosity; responsibility; and Buddhist beliefs. This cultural knowledge is more abstract and detailed. Individuals with ASD have difficulty understanding, comprehending, and generalising the skills that they learn in a number of settings. This reflects poorly on them and causes them distress when they are unable to resolve the issue. Individuals with ASD have difficulty comprehending and generalising the skills that they learn in a number of settings. This reflects poorly on them and causes them distress when they are unable to resolve the issue.

The cultural knowledge of Thai tradition that both groups mentioned, which impacts teaching social skills, was included:

Grandparents always teach a child. “If you do bad things, you will go to hell”. That child doesn’t understand what that means. Has grandma ever been in hell? It is so abstract. On the other hand, they should teach the child that if you do bad things, what is the consequence? It is not heaven or hell. This example shows how Thai culture affects children. (Nurse5: FG3)

The adolescents and their caregivers mentioned Thai culture in terms of respect for seniors, alternative opinions, social etiquette, and parenting style. Many caregivers believe their adolescents don’t understand the value of elder respect. Their adolescents behave inappropriately around them, including disputing when the elders teach them with strong words and only concentrating on right and wrong. They lacked respect for others. Thus, caregivers suggested that a social skills programme could include a session training caregiver and their adolescents on how to handle and model interactions and respectful communication with older adults.

I grew up in a different way from them [teenagers]. I’m not getting used to that. They [teenagers] don’t respect adults. Some adolescents say, for example, Hey, what’s up? and then call their friend’s father or mother’s name. Actually, parents can be your friends, but you should respect them too. In Thai culture, I don’t see them saluting adults or admitting when they do something wrong. They are quite aggressive. (Caregiver of female aged 10 years, previously attended SSI)

Additionally, the Thai culture has a suite of politeness languages based on a person’s age, family, and occupation. For instance, in the hierarchy language, if you call a person by name, you should use the courteous terms “Pee” (for the elderly) or “Nong” (for the young). The HPs identified that certain adolescents with ASD have trouble with these hierarchies. They are unaware of the value of polite language or the consequences of not using it.

##### Method:

Assertiveness and praise were the methods that both groups mentioned for improving the adolescents’ social skills. Caregivers frequently discussed the generational divide between parents and teenagers. Traditionally, in Thai culture, being passive is considered polite, and many caregivers will have grown up with these characteristics and attitudes in their behaviour. Adolescents today, however, are perceived as being more assertive than in previous generations, and so caregivers can lack an appropriate level of assertiveness with their adolescents. As a result, the caregivers suggested that a social skills programme could helpfully include assertiveness training and activities designed to practise being more assertive in communication and interactions with their adolescents.

I think Thai society has some difficulty because we don’t openly express our feelings. That makes us feel hard to interpret emotions. Unlike in Western countries where emotions are clearly expressed, it is hard for our children here. Currently, I observe that my child attempts to express his feelings, but it is still difficult because we, as parents, don’t express our feelings either. He lacks examples to follow. (Caregiver of male aged 12 years, not attended SSI)

The HPs mentioned that praise and therapeutic and comforting touch are important to encouraging the good behaviour of adolescents. However, in Thai, parents are not familiar with this method. Some parents thought that it might have spoiled their adolescents.

Thai culture is different from Western culture. In Western cultures, people hug, touch, and praise openly. Regarding praise and appreciation, mothers often ask me if they should praise their children. They don’t really understand why they need to praise because they believe it is their child’s duty to do things. Parents are also afraid that their child might become spoiled. (Nurse5; FG3)

##### Goal:

In relation to the content component, HPs discussed the social content that should be considered in the provision programme. They mentioned that the goal of a programme should include a cultural component to teach adolescents with ASD and their caregivers about the cultural challenges in social skills and how to prepare their adolescents for it, so that they can better understand and help their adolescents.

I would like to incorporate Thai customs and traditions into the programme. As we discussed, there are some positive aspects in Thai culture. We are sympathetic and we care about society. This aspect has both positive and negative implications. We should incorporate training for ASD. (Nurse6; FG3)

### Impact of the COVID-19 pandemic

This theme was related to both groups’ discussions about the impact of COVID-19 on adolescents with ASD’s social skills development, behaviour problems, and the transformation of training services. Due to COVID-19 pandemic restrictions, many adolescents with ASD had their social skills intervention postponed, according to both groups. They have been able to receive online treatment (e.g., telemedicine, individual counselling) since the COVID-19 pandemic began. This COVID-19 pandemic not only disrupted services but also contributed to an increase in behavioural issues since some adolescents spent an excessive amount of time on social media, less time on outdoor activities, and less time socialising with their friends in person.

Due to COVID-19, the programme [social skills intervention] has been suspended from last year until now. If we could attend those group sessions, it would be great because this session trains him to coexist with other people in society. There was a total of 2 cancelled sessions. (Caregiver of male aged 15 years, not attended SSI)

They kept playing computer games all day without any friends, staying quietly at home without socialisation. If we just left them, they would become even more withdrawn. We observed many children like this during COVID-19 because they all stayed at home for a long period of time. (Child psychiatrist: FG1)

Furthermore, both groups agreed on the value of online training. It sought to increase adolescents’ and families’ chances of receiving support services (HPs) and monitoring their adolescents ‘s behaviour during the epidemic (caregivers and their adolescents). Some adolescents and caregivers suggest using online lessons to practise social skills, such as simulating social activities prior to practising with others or providing an online factsheet on social skills procedures that they could access after receiving the training. In contrast, HPs considered that online methods of teaching were ineffective for developing social skills in certain areas, for example, teaching facial expressions and group activities. Thus, the online training session might be integrated into a social skills programme.

The following excerpt is taken from a conversation where both the adolescents and caregivers agreed about the role of games as an effective learning strategy during the COVID-19 pandemic.

All children like playing games, especially multiplayer games. We can do that online during the COVID-19 pandemic (Male aged 12 years, not attended SSI)

What my son just said was interesting. There might be a central platform or area for them to pick up their favourite character. In the game, you won’t see them physically work, but they will get used to each other. It is hard for these kids to get used to some new things. To ask them to work with newcomers immediately is difficult because they are very shy. However, if we introduce them with a game, then we go further to some activity. It sounds better. (Caregiver of male aged 12 years, not attended SSI)

## Themes pertaining to HPs

The following section presents the themes that were unique to the HPs focus groups. Three main themes were constructed from the HPs data including (i) Assessment of and tailoring to the priorities of adolescents with ASD, (ii) Training and education needs of HPs and (iii) Impact of workload on provision of a programme.

### Assessment of and tailoring to the priorities of adolescents with ASD

This theme emphasises the importance of assessing abilities, training needs, and tailoring the programme for adolescents with ASD and their caregivers before conducting the training. All of the HPs in the focus groups agreed that the components of a programme could be tailored to meet the unique needs of adolescents with ASD, including social skills difficulties, the developmental stage of adolescents, and the specific characteristics of the participants within a homogenous group. According to the experiences of HPs, the existing social skills programme offers training in social skills for adolescents with ASD in all areas, but the adolescents did not improve in all social skills. The HPs perceived that if they could assess training needs and prioritise the most important social skills problems based on adolescents’ development, the training could be better tailored to their needs and problems.

We solve all of the problems and train [adolescent’s with] ASD for the most serious ones. It is hard to train all of them. We should determine which issues are urgent so that he can coexist with his friend and society. (Psychologist: FG3)

The HPs discussed that a degree of homogeneity or commonality across group members is important when conducting a social skills programme. The group members could have similar abilities, age range and cognitive ability (IQ) and thus, the HPs can set the appropriate activity and goal of training suited for each group.

The homogenous membership group is very important, both in age range and common type. IQ is important too. The high function definition is not the same. Sometimes, we use IQ. Otherwise, we use social skills, which are hard to identify. For those teenagers’ programmes, we should use the same IQ range for good training. (Child psychiatrist: FG1)

### Training and education needs of HPs

This theme related to the importance of training and education for HPs to provide and deliver social skills programmes. According to the experience of HPs, they are the programme’s trainers, and implementing these interventions requires specialised knowledge, such as applied behaviour analysis (ABA; intensive behavioural interventions). In order to deliver the programme, HPs with limited expertise require assistance with education and training.

Yes, that’s right. The social skills problem is based on a lack of verbal communication. I am unable to train by myself. In some cases, his [adolescents with ASD] social skills are fine, but his verbal communication has dropped. Thus, the trainer’s skills and experience are very important. Our team members are just rookies. It would be good if we could get some more knowledge training, such as ABA. (Psychologist2: FG2)

### Impact of workload on the provision of a programme

This theme related to HPs’ perceptions that their routine workload often prevents them from getting the time to develop and improve the quality of social skills programmes. The HPs mentioned that they gave service to a high number of patients. In response to the needs of working parents, HPs also stated that they have tried to provide a weekend social skills training programme. However, this is often not possible because the HPs feel overloaded and tired with their routine work, and the government does not provide additional support for seven days working. Improved employee rotation by rescheduling work to receive two days off per week as usual and improved compensation for staff were articulated as ways that could help address these issues.

The staff may earn an overtime fee of 40 THB per hour [which corresponds to a low amount in Thai currency], excluding travel costs. This arrangement affects their families’ time, children, and health. The government always promotes the idea that one’s work and life schedule must be balanced, but there is no other option. We won’t get any cooperation because every staff member works so hard and often feels tired. (Psychologist1: FG1)

Let’s say that if we skip the compensation issue, we may reallocate the working schedule. We work five days. No one wants to work on the sixth day if there are no urgent cases. We may shift the working days so that each staff member still has two consecutive days off. (Nurse: FG1)

## Theme of adolescents with ASD and their caregivers

The themes expressed by the adolescents with ASD and their caregivers are presented in the following section. One main theme was constructed, including.

### Stigma reduces attendance at the programme

This theme was related to the stigma that adolescents with ASD perceive and had an effect on participants’ willingness to participate in the training programme. They asserted that disclosing their illness and attending to treatment at the psychiatric hospital was detrimental to their health due to the fact that their friends did not fully understand, and some teased them about this. Similarly, their parents believed that they should conceal their child’s illness from others, as they did not want their adolescents to feel different from other people. They are unwilling to attend treatment because they do not wish to be associated with a label that may generate stigma. However, if a programme is running on weekends or during a school break, they prefer to attend. Thus, conducting the training on a day when school is not in session may encourage more parents and their adolescents to participate in the programme.

I don’t tell anyone about the autism of my child. I want him to live like a normal person. Also, I don’t know what else I should tell the teacher. It is either personal leave or sick leave. That is difficult because I want him to be like a normal child with a normal reason. (Caregiver of male aged 12 years; previously attended SSI).

For my kid and me, weekends are better than weekdays. That’s great. My child doesn’t take any time off for illness [they tell teachers that they are sick rather than follow up with the doctor]. He [the adolescent] doesn’t want to be late for class. His friends tease him. The problem is that he is absent from class. The main point is that he doesn’t want his friend to know. (Caregiver of male aged 18 years; previously attended SSI)

## Discussion

The purpose of this study was to gain a better understanding of the experiences, expectations, and cultural adaptations required for an outpatient SSI programme for adolescents with ASD in Thailand, and by exploring the barriers and supportive factors to implementing such a programme. The study findings revealed nine main themes from HPs and seven main themes from adolescents with ASD and their caregivers regarding the need to improve social skills, the important feature that could be added to a social skills programme, and the cultural component that should be considered in the programme.

### The requirements of SSIs for Thai adolescents with ASD

According to stakeholders the components which are considered important in social skills programmes include the person who delivers the intervention, social skills outcomes, learning strategies, assessments, and tailoring needs in each family and Thai culture.

Parental involvement in a programme is important for improving the adolescent’s social skills and for continuing their treatment after attending the intensive clinical programme at the hospital, because parents can act as a coach by providing opportunities for social skills practise in an adolescent’s daily life. This option may be used to address the limitation of generalisation of the skills acquired during group training [67, 68]. In a reflection piece on the PEERS social skills programme translated into French. Vuattoux et al. (2021) also identified the importance of parental involvement in the maintenance of improvements in adolescents (aged 15–18 with ASD without ID) on social responsiveness, social knowledge, and social reciprocity [69]. Parents acted as agents of reinforcement and generalisation of the acquired knowledge. There was consistency in the outcomes of SSI for Dutch pre-adolescents (10–12 years with ASD; n = 120). Dekker et al. (2017) revealed the benefits of training parents in the generalisation of skills, social skills, and social functioning of pre-adolescents at home [70]. This study compared the SSI with the SSI that had added parent and teacher involvement (e.g., training parents in 8 sessions and one meeting and five phone contacts with the teacher). As a result, parents could be involved in the SSI and help with practicing, encouraging, and generalising social skills in their adolescents.

Support information on adolescents’ symptoms, social skills deficits, and practising coaching skills were important for caregivers, because such information can shape a caregiver’s attitude on their adolescent’s diagnosis and how they care for the adolescent [71]. This finding further supports the idea that parents need education to care for their adolescents [72, 73]. An et al. (2020) interviewed Kazakhstani caregivers of ASD adolescents (n = 17) to explore their perspectives on their adolescent’s education and social support [72]. These parents require a variety of interventions to assist their adolescents in enhancing the adolescent’s communication, social, occupational, and life skills. In addition, emotional support for parents (e.g., counselling) was mentioned because managing behavioural problems and caring for children can be stressful for the caregivers. This is important because previous reviews of research amongst Asian parents has highlighted that managing behavioural problems and caring for children can be stressful [71, 74]. Shorey et al. (2020) conducted a qualitative meta-analysis (N = 44) on the experiences and needs of Asian parents of children with ASD, which identified parents who were experiencing negative emotions (e.g., depression) and stress due to their children’s behavioural issues and the lack of information and abilities among HPs [71].

Specific social communication skills, problem-solving skills, emotional recognition, and developing a solid theory of mind are frequently challenging for Thai adolescents with ASD and have a significant impact on their relationships with others. A programme for social skills should train these adolescents specifically in these skills, as opposed to social skills in general. These social skills are necessary for adolescents with ASD to develop relationships with others. Swedish teenagers with a neurodevelopmental disorder [(NDD) e.g., ASD, Attention deficit disorder] agreed on the necessity of these skills [75]. Leifler et al. (2020) conducted a randomised controlled trial of group social skills training in a school context. The objective was to instruct these adolescents (aged 17 to 20; n = 20) in social communication and emotional recognition [75]. Interviews indicated that these teenagers benefitted from this instruction, which enables them to know how to engage with their classmates and others. Consequently, these specific abilities are beneficial for teaching and practising with adolescents with ASD.

The learning strategies of incorporating multimedia into SSI, peer modelling, group activities, and homework assignments are important strategies for teaching and learning key social skills because these strategies encourage adolescents to be understanding, have a role model and practise social behaviour in daily life. The benefits of having peer role models were evident in a peer-mediated programme with didactic instruction, modelling, role-play, and homework in adolescents [(Mean aged 14.72 with ASD); (n = 62)] [76]. Dean et al. (2020) found that peer mentorship (assistance in doing and joining social activities) had a positive impact on adolescents’ social engagement, quality interpersonal relationships, and decreased social anxiety in the school setting [76]. In addition, Ahmad et al. (2019) reported the finding from Malaysian’ professionals (n = 30) that individuals with ASD required multimedia tools (e.g., a graphical user interface, text, images, sound, animation, and video) to encourage their learning about communication, interactions, and engagement with other people [77]. As a result, in the SSI, these learning strategies may be advantageous to provide on the programme.

### The impact of Thai cultural and social expectations on developing and delivering SSI

Cultural considerations include the need for social knowledge of Thai culture; encouraging assertiveness and praising parents’ skills; implementing a programme at a time that does not interrupt academic achievement; and renaming the programme. The participants in this study mentioned that these elements influence therapeutic relationships, thereby increasing adolescents’ or caregivers’ engagement in a programme and assisting adolescents in maintaining relationships with members of their social communities.

Social knowledge of Thai culture showed in values on respect for the elderly as well as morality, obedience, generosity, responsibility, and Buddhism. According to participants in this study, this social knowledge is important but complex and difficult to learn for adolescents with ASD. Vibulpatanavong, (2017) also mentioned in interviews with Thai participants (e.g., parents of children with ASD and special educators; n = 10) that Thai culture (e.g., the Thai greeting) is valuable, but educating individuals with ASD is more challenging and requires more construction [78]. Therefore, a social skills programme for adolescents with ASD could include cultural topics to assist them in learning more about their social world and building stronger relationships. Furthermore, social knowledge can affect the frequency of engagement in a programme, which is reflected in the adaptation of PEERs in Asian countries. The PEERs Chinese version [79] maintained the “get together” session (e.g., going to lake, playing sport), while the PEERs Hebrew version [80] adapted the activities to common Israeli ones (e.g., touring the house). The results showed that the frequency of engaging into this session was higher amongst Hebrew participants than Chinese participants. Thus, social knowledge could be added to the programme. Future research may be able to determine how to educate these adolescents on this complex social knowledge.

In addition, assertiveness and praise were other methods that could be considered in Thai culture. These methods of teaching are important to gain more positive social behaviour, but they’re not familiar to Eastern parents. The Chinese parents’ perspective on praising is that “more praise for their children means more spoiling them” [81], which is reflected in a similar view held by Thai parents. According to the Lau (2012) study, which conducted interview HPs (n = 24) to understand the need for cultural adaptation in parenting programmes for Chinese American and Latino parents, this issue was faced, and solutions were offered. HPs in this study addressed these concerns by translating the concept of praise as reinforcement, which can encourage positive behaviour in their adolescents and reflect parents’ feelings when they received reinforcement [81]. These techniques were useful to the studied immigrant parents. As a result, future research may make these techniques applicable to Thai parents.

Academic achievement is more important to parents in a number of Asian countries than their adolescents ‘s social skills [82, 83], while the HPs prioritise the SSI over academic achievement. These perceptions might affect therapeutic relationships and collaboration in SSI. These results showed in the Yoo et al. (2014) study that the completion rate of homework in PEER’s Korean version was less than 50% due to the fact that the study’s participants (n = 47) attended extra activities such as studying in private tutoring institutions [84]. Therefore, according to the HPs in this study, managing parents’ expectations and acceptance of their child’s illness and implementing a programme that does not interrupt academic achievement may allow for more effective collaboration on the training programme.

### Barriers and facilitators to implementing SSIs

Throughout the COVID-19 pandemic, the majority of participants postponed social skills training in favour of online treatment (e.g., telemedicine). It had an effect on the social skills of adolescents with ASD. The caregivers perceived online treatments to be beneficial for monitoring their adolescents ‘s behaviour but not for social skill development. Similarly, the HPs stated that this type of online training is ineffective for teaching certain areas of social skills (e.g., facial expressions). As a result, a programme could use online treatment as a part of the programme. For example, Bross et al. (2022) designed a SSI for young adults with ASD (aged 17–26 years, n = 8) that included both face-to-face social skills training and online instructional modules. The results indicated that the young adults with ASD had significantly increased their knowledge compared to the baseline scores [85].

Adolescents with ASD and their caregivers have negative experiences with public stigma against their diagnosis (e.g., at school). Some adolescents with ASD face teasing or bullying as a result of a widespread lack of knowledge about and understanding of the disorder [86]. As a result, caregivers refrain from disclosing their adolescents ‘s diagnoses out of fear of discrimination [87] which affecting to receive the treatment programme. A meta-analysis (n = 44) of Shorey et al. (2020) found that Asian caregivers (24 studies) had social stigma against mental health illness [71]. In addition, Lim et al. [88]’s survey study found that Taiwanese caregivers who had high stigma scores had an effect on their adolescents’ (with ASD, aged 10–16; n = 76) social experiences. These adolescents were less interested in interacting with people at school. Thus, a programme could facilitate caregivers’ efforts to manage stigma [88, 89].

Barriers unique to HPs include: the need for specific training and education to be able to deliver a programme that suitably addresses their clients’ needs and priorities and the impact of delivering a programme on workload. The HPs highlighted that the workload and trainer competence were the barriers to the implementation of the programme. These are similar to the barriers for implementing educational interventions for ASD [90]. As a result, a programme could provide training for trainers [91] and admiration could support the compensation for HPs. Additionally, they suggested that if the policy facilitates compensation and relocation of staff (e.g., rescheduling work to receive two days off per week as usual), these benefits can help facilitate the implementation of a social skills programme.

## Limitations

To our knowledge, this is the first study that has explored the SSI requirements and essential social knowledge of Thai culture for Thai adolescents with ASD, based on input from stakeholders. These findings could contribute to the cultural adaptation SSI in Thailand. However, the study has several limitations. Firstly, this study does not represent the views of the teachers and peers who were frequently mentioned in the interviews. Friendships with peers and teachers are important for adolescents with ASD [92]. Future studies could include the perspective of these participants. Secondly, during the interviews, the interviewers were able to identify signs of discomfort in adolescents with ASD. This study employed the paired interview, which helps adolescents with ASD feel comfortable communicating with other people and has a protocol for managing distress [59]. However, this study found that one adolescent with ASD required privacy to tell some stories without their parents. As a result, the interview separated the session from their caregiver for 15 min, which allowed the adolescents to describe their experience. Therefore, it is essential that the interview process evaluate the unique needs and capabilities of adolescents with ASD [61].

## Conclusion

Adolescents with ASD experience social skills problems, which affect several areas of their lives, for example, their relationships, mental health, and daily functioning. From the perspective of stakeholders, these adolescents require a programme that encourages their social skills and strengthens the role of their caregivers as co-therapists. Outpatient SSIs could consist of specific knowledge and skills for improving social skills in adolescents with ASD, knowledge and coaching skills for their caregivers, practising social skills with some daily activities, and using an interesting, practical, and self-practiced learning strategy. The distinctiveness of Thai culture could be incorporated into the programme’s instructional content, method and implementation. These cultural elements can enhance cooperation within the training programme, the rapport between patients and therapists, and the efficacy of social skills outcomes. Training for the trainers and compensation for the HPs could reduce barriers to the programme’s implementation. In addition, the stigma of attending training at a psychiatric hospital, and the limited availability of treatment during the COVID-19 pandemic should also be taken into account.

### Electronic supplementary material

Below is the link to the electronic supplementary material.

**Supplementary Material 1**: Paired Depth Interview Guideline

**Supplementary Material 2**: Focus Group Interview Guideline

## Data Availability

Due to ethical and legal restrictions concerning the confidentiality of study participants, the generated and analysed datasets are not available to the public.

## Abbreviations

HPS: Healthcare professionals
SSIs: Social skills interventions
ASD: Autism Spectrum Disorder
