# Supplementary material for: Social skills interventions for Thai adolescents with Autism Spectrum Disorder (ASD): a qualitative study of the perceptions and experiences of Thai adolescents, their caregivers and healthcare professionals
Source: Int J Ment Health Syst. 2024 Jan 2;18:1. doi: 10.1186/s13033-023-00617-3 (PMC10763348; doi:10.1186/s13033-023-00617-3)
Supplement: Supplementary file 1 — Supplementary Material 1: Paired Depth Interview Guideline [file 13033_2023_617_MOESM1_ESM.docx]

**Paired depth interview guideline**

**(adolescents with ASD and their care givers)**

**Introduction**

- Explanation of ethics, consent and confidentiality of interview and analysis.
- Explanation of objective of acceptability sub study.
- Structure and duration of the interview.
- Any questions?

**Experiences of ASD and its impact**

1. Can you tell me about how long you/your son/daughter has been diagnosed with ASD?
2. Can you tell me more about the events leading up to receiving a diagnosis of ASD?
3. What is life like for you now’ (separate this out into asking the adolescent and also the family member)?
4. Can you tell me about any difficulties that you/your son/daughter have had with socializing?
5. What kinds of support have you received for helping with this?

**Experiences of social skills intervention**

1. Have you ever been part of a social skills programme?
2. Can you tell me what the term ‘social skill intervention’ means to you?
3. What’s most important to you in relation to social skills intervention?
4. What’s was most helpful/unhelpful?
5. How important is this to you? Do you think this intervention that you receive helps build your social skills?

**Expectations for an outpatient base social skills intervention**

1. What’s social skills are most problematic for you and your child and for which you would like help to manage?

(e.g. 1) social communication (making friend, two –way communication, appropriate use humour, understand and applying the rules of good sportsmanship, 2) social competence (cooperation, assertion, self-control) 3) social cognition (theory of mind, emotional recognition)

1. What features do you think should be included in a social skills training for adolescents with ASD? (number of sessions, who delivery, social skills outcome, component of intervention)?

*Prompts: From the previous review found that the element on the social skill intervention could include:*

*2.1 What kinds of information or education would you want as part of a social skills training programme?*

*2.2 Who would be the key person to help deliver social skills training programme? (e.g. parents, health professionals (which ones), peer adolescents?)*

*2.3 Who should be involved in the programme itself? (e.g. staff, parent, peers)*

*2.4 How long do you think the programme should last for? How many sessions would you like this to be? What days specifically should it run on? (e.g. workday, weekend)*

*2.5 What is the best setting for training? (e.g. hospital (inpatient or outpatient), school, combined, elsewhere in community)?*

*2.6 Do you think technology (e.g. virtual reality, software, presentation) should include on the programme?*

*2.7 Is there anything else I haven’t covered that you think would be important in a social skills training programme?*

*In all questions here probe: Would that be something that is important to you? Work well for you? [if yes, why; if no, why not?*

**Social expectations in Thai culture**

1. What the social expectation in Thai culture is effect on you? (e.g. responsibility, good children)
2. What’s the cultural norm for Thai ’s adolescents?
3. What’s the cultural norm for people who are the good person in your community? (passive, caring, assertive)

**Ending**

We come to end my question.

Is there anything else you would like to talk about?

Thank you the participations.
