# Supplementary material for: Social skills interventions for Thai adolescents with Autism Spectrum Disorder (ASD): a qualitative study of the perceptions and experiences of Thai adolescents, their caregivers and healthcare professionals
Source: Int J Ment Health Syst. 2024 Jan 2;18:1. doi: 10.1186/s13033-023-00617-3 (PMC10763348; doi:10.1186/s13033-023-00617-3)
Supplement: Supplementary file 2 — Supplementary Material 2: Focus Group Interview Guideline [file 13033_2023_617_MOESM2_ESM.docx]

**Focus group guideline**

**(Healthcare professionals)**

**Introduction**

- Explanation of ethics, consent and confidentiality of interview and analysis.
- Explanation of objective of acceptability sub study.
- Structure and duration of the interview.
- Any questions?

**Experience of caring for adolescents with ASD and their families**

1. what is your role in the hospital and what’s your involvement in the care of adolescents with ASD and their families?
2. What would you say are the main difficulties that adolescents with ASD and their families experience?
3. What kind of support do you offer to adolescents and their families for helping with social skills development?

**Experience of social skill intervention**

1. Can you tell me what the term ‘social skill intervention’ means to you?
2. How helpful/useful do you think these programmes are for adolescents with ASD and their families?
3. From your perspective, what’s was most helpful/unhelpful?
4. How important is this to your patient? Do you think this intervention that you give for patients can build their social skills? (How successful it’s been, any challenges they’ve seen or experienced in relation to the programme? )

**Expectations for an outpatient base social skills intervention**

1. What’s social skills are most problematic for your patient and which you would like help to manage?

*(e.g. 1) social communication (making friend, two –way communication, appropriate use humour, understand and applying the rules of good sportsmanship, 2) social competence (cooperation, assertion, self-control) 3) social cognition (theory of mind, emotional recognition)*

1. What features do you think should be included in a social skills training for adolescents with ASD? (number of sessions, who delivery, social skills outcome, component of intervention)?

*Prompts: From the previous study found that the element on the social skill intervention could including:* (Would that be something that is important to you? Work well for you? [if yes, why; if no, why not?)

*2.1 What kinds of information or education would you want as part of a social skills training programme?*

*2.2 Who would be the key person to help deliver social skills training programme? (e.g. parents, health professionals (which ones), peer adolescents?)*

*2.3 Who should be involved in the programme itself? (e.g. staff, parent, peers)*

*2.4 How long do you think the programme should last for? How many sessions would you like this to be? What days specifically should it run on? (e.g. workday, weekend)*

*2.5 What is the best setting for training? (e.g. hospital (inpatient or outpatient), school,*

*combined, elsewhere in community)?*

*2.6 Do you think technology (e.g. virtual reality, software, presentation) should include on the programme?*

*2.7 Is there anything else I haven’t covered that you think would be important in a social skills training programme?*

*In all questions here probe: Would that be something that is important to you? Work well for you? [if yes, why; if no, why not?*

**Cultural in Thai culture**

1. What is the Thai cultural issue should be considerate when adapted social skills intervention?
2. What’s the social activity which important for adolescent in Thailand?
3. Do you think slang word is important to communicate with other people?
4. What’s do you think about “humor”? Is it important?
5. What the social expectation in Thai culture is effect on your patient? (e.g. responsibility, good children)

**Barriers to implementing outpatient base social skills intervention**

1. What do you think might affect or stop the intervention from being delivered/working?
2. Have you ever had any issue or problems with delivery this intervention?
3. What’s the main problems that we can’t teach the social skills in adolescent with ASD?

**Facilitators to implementing outpatient base social skills intervention**

1. What is the facilitator when implement this intervention?
2. Who’s the key success to implement this programme?
3. Do you have any recommendations for implementation of a social skills training programme in out patient’s clinic?

**Ending**

We come to end my question.

Is there anything else you would like to talk about?

Thank you the participations.
